# Obesity and kidney disease: hidden consequences of the epidemic

**DOI:** 10.4155/fsoa-2016-0081

**Published:** 2017-02-06

**Authors:** Csaba P Kovesdy, Susan L Furth, Carmine Zoccali, on behalf of the World Kidney Day Steering Committee

**Affiliations:** 1 Division of Nephrology, Department of Medicine, University of Tennessee Health Science Center, Memphis, TN, USA; 2 Nephrology Section, Memphis VA Medical Center, Memphis, TN, USA; 3 Department of Pediatrics, Perelman School of Medicine at the University of Pennsylvania, Philadelphia, PA, USA; 4 CNR – IFC Clinical Epidemiology & Pathophysiology of Renal Diseases & Hypertension, Reggio Calabria, Italy

**Keywords:** chronic kidney disease, kidney cancer, nephrolithiasis, obesity, prevention

## Abstract

Obesity has become a worldwide epidemic, and its prevalence has been projected to grow by 40% in the next decade. This increasing prevalence has implications for the risk of diabetes, cardiovascular disease and also for chronic kidney disease. A high BMI is one of the strongest risk factors for new-onset chronic kidney disease. In individuals affected by obesity, a compensatory hyperfiltration occurs to meet the heightened metabolic demands of the increased body weight. The increase in intraglomerular pressure can damage the kidneys and raise the risk of developing chronic kidney disease in the long-term. The incidence of obesity-related glomerulopathy has increased tenfold in recent years. Obesity has also been shown to be a risk factor for nephrolithiasis, and for a number of malignancies including kidney cancer. This year the World Kidney Day promotes education on the harmful consequences of obesity and its association with kidney disease, advocating healthy lifestyle and health policy measures that make preventive behaviors an affordable option.

In 2014, over 600 million adults worldwide, 18 years and older, were obese. Obesity is a potent risk factor for the development of kidney disease. It increases the risk of developing major risk factors for chronic kidney disease (CKD), like diabetes and hypertension, and it has a direct impact on the development of CKD and end-stage renal disease (ESRD). In individuals affected by obesity, a (likely) compensatory mechanism of hyperfiltration occurs to meet the heightened metabolic demands of the increased body weight. The increase in intraglomerular pressure can damage the kidney structure and raise the risk of developing CKD in the long-term.

The good news is that obesity, as well as the related CKD, are largely preventable. Education and awareness of the risks of obesity and a healthy lifestyle, including proper nutrition and exercise, can dramatically help in preventing obesity and kidney disease. This article reviews the association of obesity with kidney disease on the occasion of the 2017 World Kidney Day.

## Epidemiology of obesity in adults & children

Over the last three decades, the prevalence of overweight and obese adults (BMI ≥25 kg/m [1]) worldwide has increased substantially [2]. In the USA, the age-adjusted prevalence of obesity in 2013–2014 was 35% among men and 40.4% among women [1]. The problem of obesity also affects children. In the USA in 2011–2014, the prevalence of obesity was 17% and extreme obesity 5.8% among youth 2–19 years of age. The rise in obesity prevalence is also a worldwide concern [3,4], as it is projected to grow by 40% across the globe in the next decade. Low- and middle-income countries are now showing evidence of transitioning from normal weight to overweight and obesity as parts of Europe and the USA did decades ago [5]. This increasing prevalence of obesity has implications for cardiovascular disease and also for CKD. A high BMI is one of the strongest risk factors for new-onset CKD [6,7].

Definitions of obesity are most often based on BMI (i.e., weight ([kilograms] divided by the square of his or her height [meters]). A BMI between 18.5 and 25 kg/m [1] is considered by the WHO to be normal weight, a BMI between 25 and 30 kg/m [1] as overweight, and a BMI of >30 kg/m [1] as obese. Although BMI is easy to calculate, it is a poor estimate of fat mass distribution, as muscular individuals or those with more subcutaneous fat may have a BMI as high as individuals with larger intra-abdominal (visceral) fat. The latter type of high BMI is associated with substantially higher risk of metabolic and cardiovascular disease. Alternative parameters to more accurately capture visceral fat include waist circumference (WC) and a waist–hip ratio (WHR) of >102 cm and 0.9, respectively, for men and >88 cm and >0.8, respectively, for women. WHR has been shown to be superior to BMI for the correct classification of obesity in CKD.

## Association of obesity with CKD & other renal complications

Numerous population based studies have shown an association between measures of obesity and both the development and the progression of CKD (Table 1). Higher BMI is associated with the presence [8] and development [9–11] of proteinuria in individuals without kidney disease. Furthermore, in numerous large population-based studies, higher BMI appears associated with the presence [8,12] and development of low estimated glomerular filtration rate [9,10,13], with more rapid loss of estimated glomerular filtration rate over time [14] and with the incidence of ESRD [15–18]. Elevated BMI levels, class II obesity and above, have been associated with more rapid progression of CKD in patients with pre-existing CKD [19]. A few studies examining the association of abdominal obesity using WHR or WC with CKD, describe an association between higher girth and albuminuria [20], decreased glomerular filtration rate [8] or incident ESRD [21] independent of BMI level.

**Table 1.** **Studies examining the association of obesity with various measures of chronic kidney disease.**

**Study**	**Patients**	**Exposure**	**Outcomes**	**Results**	**Comments**	**Ref.**
Prevention of Renal and Vascular End-Stage Disease (PREVEND) Study	7676 Dutch individuals without diabetes	Elevated BMI (overweight and obese^†^), and central fat distribution (waist–hip ratio)	Presence of urine albumin 30–300 mg/24 h Elevated and diminished GFR	Obese + central fat: higher risk of albuminuria Obese ± central fat: higher risk of elevated GFR Central fat ± obesity associated with diminished filtration	Cross-sectional analysis	[8]
Multinational Study of Hypertensive Outpatients	20,828 patients from 26 countries	BMI and waist circumference	Prevalence of albuminuria by dip stick	Higher waist circumference associated with albuminuria independent of BMI	Cross-sectional analysis	[20]
Framingham multi-detector computed tomography (MDCT) cohort	3099 individuals	Visceral adipose tissue (VAT) and subcutaneous adipose tissue (SAT)	Prevalence of UACR >25 mg/g in women and >17 mg/g in men	VAT associated with albuminuria in men, but not in women	Cross-sectional analysis	[22]
CARDIA (Coronary Artery Risk Development in Young Adults) study	2354 community-dwelling individuals with normal kidney function aged 28–40 years	Obesity (BMI >30 kg/m^2^) Diet and lifestyle-related factors	Incident microalbuminuria	Obesity (OR: 1.9) and unhealthy diet (OR: 2.0) associated with incident albuminuria	Low number of events	[11]
Hypertension Detection and Follow-Up Program	5897 hypertensive adults	Overweight and obese BMI^†^ versus normal BMI	Incident CKD (1+ or greater proteinuria on urinalysis and/or an eGFR <60 ml/min/1.73 m^2^)	Both overweight (OR: 1.21) and obesity (OR: 1.40) associated with incident CKD	Results unchanged after excluding diabetics	[10]
Framingham Offspring Study	2676 individuals free of CKD stage 3	High versus normal BMI^†^	Incident CKD stage 3 Incident proteinuria	Higher BMI not associated with CKD3 after adjustments Higher BMI associated with increased odds of incident proteinuria	Predominantly white, limited geography	[9]
Physicians’ Health Study	11,104 initially healthy men in USA	BMI quintiles Increase in BMI over time (vs stable BMI)	Incident eGFR <60 ml/min/1.73 m^2^	Higher baseline BMI and increase in BMI over time both associated with higher risk of incident CKD	Exclusively men	[13]
Nation-Wide US Veterans Administration Cohort	3,376,187 US veterans with baseline eGFR ≥60 ml/min/1.73 m^2^	BMI categories from <20 to >50 kg/m^2^	Rapid decline in kidney function (negative eGFR slope of >5 ml/min/1.73 m^2^)	BMI >30 kg/m^2^ associated with rapid loss of kidney function	Associations more accentuated in older individuals	[14]
Nation-Wide Population-Based Study from Sweden	926 Swedes with moderate/advanced CKD compared with 998 controls	BMI ≥25 versus <25 kg/m^2^	CKD versus no CKD	Higher BMI associated with 3× higher risk of CKD	Risk strongest in diabetics, but also significantly higher in nondiabetics Cross-sectional analysis	[12]
Nation-Wide Population-Based Study in Israel	1,194,704 adolescent males and females examined for military service	Elevated BMI (overweight and obesity) versus normal BMI^†^	Incident ESRD	Overweight (HR: 3.0) and obesity (HR: 6.89) associated with higher risk of ESRD	Associations strongest for diabetic ESRD, but also significantly higher for nondiabetic ESRD	[17]
The Nord-Trøndelag Health Study (HUNT-1)	74,986 Norwegian adults	BMI categories^†^	Incidence of ESRD or renal death	BMI >30 kg/m^2^ associated with worse outcomes	Associations not present in individuals with BL <120/80 mmHg	[15]
Community-Based Screening in Okinawa, Japan	100,753 individuals >20 years old	BMI quartiles	Incidence of ESRD	Higher BMI associated with increased risk of ESRD in men, but not in women	Average BMI lower in Japan compared with Western countries	[16]
Nation-Wide US Veterans Administration Cohort	453,946 US veterans with baseline eGFR<60 ml/min per 1.73 m^2^	BMI categories from <20 to >50 kg/m^2^	Incidence of ESRD Doubling of serum creatinine Slopes of eGFR	Moderate and severe obesity associated with worse renal outcomes	Associations present but weaker in patients with more advanced CKD	[19]
Kaiser Permanente Northern California	320,252 adults with and without baseline CKD	Overweight, class I, II and extreme obesity; versus normal BMI^†^	Incidence of ESRD	Linearly higher risk of ESRD with higher BMI categories	Associations remained present after adjustment for DM, hypertension and baseline CKD	[18]
REGARDS (Reasons for Geographic and Racial Differences in Stroke) Study	30,239 individuals	Elevated waist circumference or BMI	Incidence of ESRD	BMI above normal not associated with ESRD after adjustment for waist circumference Higher waist circumference associated with ESRD	Association of waist circumference with ESRD became on-significant after adjustment for comorbidities and baseline eGFR and proteinuria	[21]

^†^Normal weight: BMI 18.5–24.9 kg/m^2^; overweight: BMI 25.0–29.9 kg/m^2^; class I obesity: BMI 30.0–34.9 kg/m^2^; class II obesity: BMI 35.0–39.9 kg/m^2^; class III obesity: BMI ≥40 kg/m^2^.

CKD: Chronic kidney disease; DM: Diabetes mellitus; eGFR: Estimated glomerular filtration rate; ESRD: End stage renal disease; HR: Hazard ratio; OR: Odds ratio; UACR: Urine albumin–creatinine ratio.

Higher visceral adipose tissue measured by computed tomography has been associated with a higher prevalence of albuminuria in men [22]. The observation of a BMI-independent association between abdominal obesity and poorer renal outcomes is also described in relationship with mortality in patients with ESRD [23] and kidney transplant [24], and suggests a direct role of visceral adiposity. In general, the associations between obesity and poorer renal outcomes persist even after adjustments for possible mediators of obesity's cardiovascular and metabolic effects, such as high blood pressure and diabetes mellitus, suggesting that obesity may affect kidney function through mechanisms in part unrelated to these complications (*vide infra*).

The deleterious effect of obesity on the kidneys extends to other complications such as nephrolithiasis and kidney malignancies. Higher BMI is associated with an increased prevalence [25] and incidence [26,27] of nephrolithiasis. Furthermore, weight gain over time, and higher baseline WC were also associated with higher incidence of nephrolithiasis [27]. Obesity is associated with various types of malignancies, particularly cancers of the kidneys. In a population-based study of 5.24 million individuals from the UK, a 5 kg/m [1] higher BMI was associated with a 25% higher risk of kidney cancers, with 10% of all kidney cancers attributable to excess weight [28]. Another large analysis examining the global burden of obesity on malignancies estimated that 17 and 26% of all kidney cancers in men and women, respectively, were attributable to excess weight [29]. The association between obesity and kidney cancers was consistent in both men and women, and across populations from different parts of the world in a meta-analysis that included data from 221 studies (of which 17 examined kidney cancers) [30]. Among the cancers examined in this meta-analysis, kidney cancers had the third highest risk associated with obesity (relative risk per 5 kg/m [1] higher BMI: 1.24; 95% CI: 1.20–1.28; p < 0.0001) [30].

## Mechanisms of action underlying the renal effects of obesity

**Figure 1.**
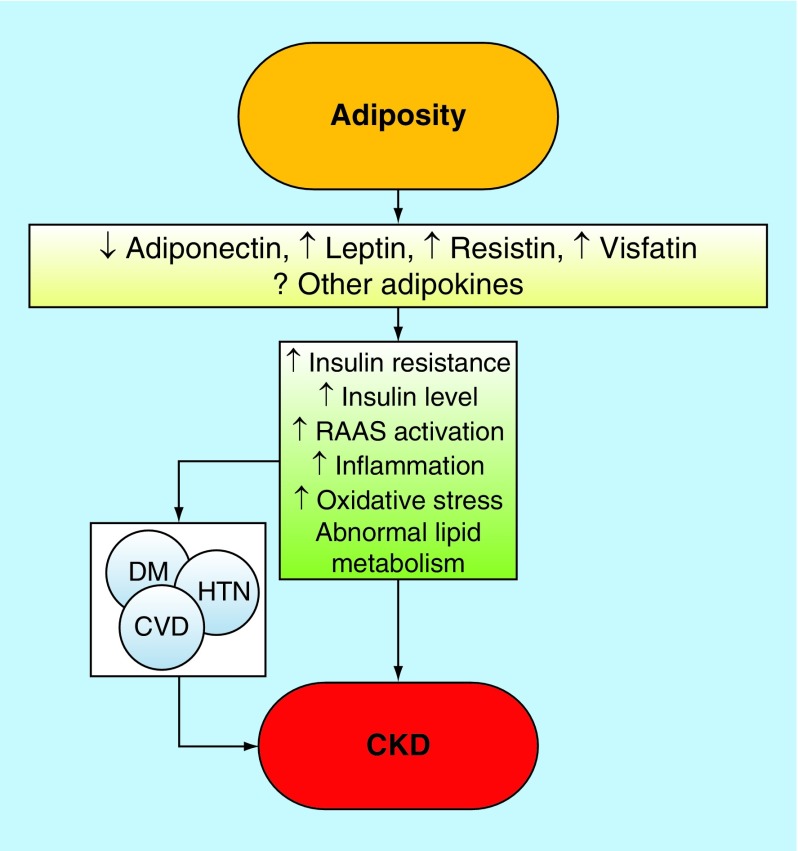
**Putative mechanisms of action whereby obesity causes chronic kidney disease.** CKD: Chronic kidney disease; CVD: Cardiovascular disease; DM: Diabetes mellitus; HTN: Hypertension.

Obesity results in complex metabolic abnormalities which have wide-ranging effects on diseases affecting the kidneys. The exact mechanisms whereby obesity may worsen or cause CKD remain unclear. The fact that most obese individuals never develop CKD, and the distinction of up to as many as 25% of obese individuals as ‘metabolically healthy’ suggests that increased weight alone is not sufficient to induce kidney damage [31]. Some of the deleterious renal consequences of obesity may be mediated by downstream comorbid conditions such as diabetes mellitus or hypertension, but there are also effects of adiposity which could impact the kidneys directly, induced by the endocrine activity of the adipose tissue via production of (among others) adiponectin [32], leptin [33] and resistin (Figure 1) [34]. These include the development of inflammation [35], oxidative stress [36], abnormal lipid metabolism [37], activation of the renin-angiotensin-aldosterone system [38] and increased production of insulin and insulin resistance [39,40].

**Figure 2.**
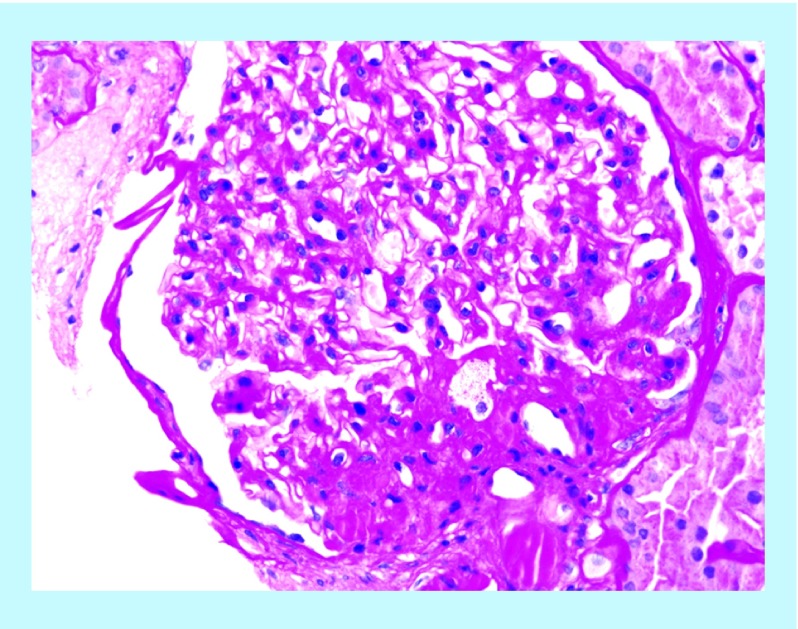
**Obesity-related perihilar focal segmental glomerulosclerosis on a background of glomerulomegaly.** Periodic Acid-Schiff stain, original magnification 400×. Courtesy of Dr Patrick D Walker, MD; Arkana Laboratories, Little Rock, AR.

These various effects result in specific pathologic changes in the kidneys [41] which could underlie the higher risk of CKD seen in observational studies. These include ectopic lipid accumulation [42] and increased deposition of renal sinus fat [43,44], the development of glomerular hypertension and increased glomerular permeability caused by hyperfiltration-related glomerular filtration barrier injury [45], and ultimately the development of glomerulomegaly [46], and focal or segmental glomerulosclerosis (Figure 2) [41]. The incidence of the so-called obesity-related glomerulopathy has increased tenfold between 1986 and 2000 [41]. Importantly, obesity-related glomerulopathy often presents along with pathophysiologic processes related to other conditions or advanced age, conspiring to result in more accentuated kidney damage in patients with high blood pressure [47] or in the elderly [14,39].

Obesity is associated with a number of risk factors contributing to the higher incidence and prevalence of nephrolithiasis. Higher body weight is associated with lower urine pH [48] and increased urinary oxalate [49], uric acid, sodium and phosphate excretion [50]. Diets richer in protein and sodium may lead to a more acidic urine and decrease in urinary citrate, also contributing to kidney stone risk. The insulin resistance characteristic of obesity may also predispose to nephrolithiasis [51] through its impact on tubular Na-H exchanger [52] and ammoniagenesis [53], and the promotion of an acidic milieu [54]. Complicating the picture is the fact that some weight loss therapies result in a worsening, rather than an improvement in the risk for kidney stone formation; for example, gastric surgery can lead to a substantial increase in enteral oxalate absorption and enhanced risk of nephrolithiasis [55].

The mechanisms behind the increased risk of kidney cancers observed in obese individuals are less well characterized. Insulin resistance, and the consequent chronic hyperinsulinemia and increased production of IGF 1 and numerous complex secondary humoral effects may exert stimulating effects on the growth of various types of tumor cells [56]. More recently, the endocrine functions of adipose tissue [57], its effects on immunity [58] and the generation of an inflammatory milieu with complex effects on cancers [59,60] have emerged as additional explanations.

## Obesity in patients with advanced kidney disease: the need for a nuanced approach

Considering the above evidence about the overwhelmingly deleterious effects of obesity on various disease processes, it is seemingly counterintuitive that obesity has been consistently associated with lower mortality rates in patients with advanced CKD [19,61] and ESRD [62,63]. Similar ‘paradoxical’ associations have also been described in other populations, such as in patients with congestive heart failure [64], chronic obstructive pulmonary disease [65], rheumatoid arthritis [66] and even in old individuals [67]. It is possible that the seemingly protective effect of a high BMI is the result of the imperfection of BMI as a measure of obesity, as it does not differentiate the effects of adiposity from those of higher nonadipose tissue. Indeed, studies that separated the effects of a higher WC from those of higher BMI showed a reversal of the inverse association with mortality [23,24]. Higher muscle mass has also been shown to explain at least some of the positive effects attributed to elevated BMI [63,68]. However, there is also evidence to suggest that higher adiposity, especially subcutaneous (nonvisceral) fat, may also be associated with better outcomes in ESRD patients [62]. Such benefits may indeed be present in patients who have very low short-term life expectancy, such as most ESRD patients [69]. Indeed, some studies that examined the association of BMI with time-dependent survival in ESRD have shown a marked contrast between protective short-term effects versus deleterious longer term effects of higher BMI [70]. There are several putative short term benefits that higher body mass could portend, especially to sicker individuals. These include a benefit from the better nutritional status typically seen in obese individuals, and which provides better protein and energy reserves in the face of acute illness, and a higher muscle mass with enhanced antioxidant capacity [63] and lower circulating actin and higher plasma gelsolin levels [71], which are associated with better outcomes. Other hypothetically beneficial characteristics of obesity include a more stable hemodynamic status with mitigation of stress responses and heightened sympathetic and renin–angiotensin activity [72]; increased production of adiponectines [73] and soluble TNF-α receptors [74] by adipose tissue neutralizing the adverse effects of TNF-α; enhanced binding of circulating endotoxins [75] by the characteristically higher cholesterol levels seen in obesity; and sequestration of uremic toxins by adipose tissue [76].

## Potential interventions for management of obesity

Obesity engenders kidney injury via direct mechanisms through deranged synthesis of various adipose tissue cytokines with nephrotoxic potential, as well as indirectly by triggering diabetes and hypertension, in other words, two conditions that rank among the strongest risk factors for CKD. Perhaps due to the survival advantage of obesity in CKD, the prevalence of end stage kidney disease is on the rise both in the USA [77] and in Europe [78]. Strategies for controlling the obesity-related CKD epidemic at population level and for countering the evolution of CKD toward kidney failure in obese patients represent the most tantalizing task that today's health planners, health managers and nephrologists face.

### Countering CKD at population level

Calls for public health interventions in the community to prevent and treat CKD at an early stage have been made by major renal associations, including the International Society of Nephrology, International Federation of the Kidney Foundation, the European renal association (ERA-EDTA) and various national societies. In the USA, Healthy People 2020, a program that sets 10-year health targets for health promotion and prevention goals, focuses both on CKD and obesity. Surveys to detect obese patients, particularly those with a high risk of CKD (e.g., hypertensive and/or diabetic obese people) and those receiving suboptimal care to inform these patients of the potential risk for CKD they are exposed to, is the first step toward developing public health interventions. Acquiring evidence that current interventions to reduce CKD risk in the obese are efficacious and deployable, is an urgent priority to set goals and means for risk modification. Appropriate documentation of existing knowledge distilling the risk and the benefits of primary and secondary prevention interventions in obese people, and new trials in this population to fill knowledge gaps (see below) are needed. Finally, surveillance programs that monitor progress on the detection of at-risk individuals and the effectiveness of prevention programs being deployed [79] constitute the third, fundamental element for establishing efficacious CKD prevention plans at population level.

A successful surveillance system for CKD has already been implemented in some places such as the UK [80]. A campaign to disseminate and apply Kidney Disease Outcomes Quality Initiative (K-DOQI) CKD guidelines in primary care within the UK National Health Service was launched. This progressively increased the adoption of K-DOQI guidelines and, also thanks to specific incentives for UK general physicians to detect CKD, led to an impressive improvement in the detection and care of CKD, in other words, better control of hypertension and increased use of angiotensin-converting enzyme and angiotensin receptor blockers [80]. This system may serve as a platform to improve the prevention of obesity-related CKD. Campaigns aiming at reducing the obesity burden are now at center stage worldwide and are strongly recommended by the WHO and it is expected that these campaigns will reduce the incidence of obesity-related complications, including CKD. However obesity-related goals in obese CKD patients remain vaguely formulated, largely because of the paucity of high-level evidence intervention studies to modify obesity in CKD patients [81].

### Prevention of CKD progression in obese people with CKD

Observational studies in metabolically healthy obese subjects show that the obese phenotype unassociated with metabolic abnormalities *per se* predicts a higher risk for incident CKD [82] suggesting that obesity *per se* may engender renal dysfunction and kidney damage even without diabetes or hypertension (*vide supra*). In overweight or obese diabetic patients, a lifestyle intervention including caloric restriction and increased physical activity compared with a standard follow-up based on education and support to sustain diabetes treatment reduced the risk for incident CKD by 30%, although it did not affect the incidence of cardiovascular events [83]. Such a protective effect was partly due to reductions in body weight, HbA1c and systolic BP. No safety concerns regarding kidney-related adverse events were seen [83]. In a recent meta-analysis collating experimental studies in obese CKD patients, interventions aimed at reducing body weight showed coherent reductions in blood pressure, glomerular hyper-filtration and proteinuria [81]. A thorough *post hoc* analysis of the REIN study showed that the nephron-protective effect of angiotensin-converting enzyme (ACE) inhibition in proteinuric CKD patients was maximal in obese CKD patients, but minimal in CKD patients with normal or low BMI [84]. Of note, bariatric surgical intervention has been suggested for selected CKD and ESRD patients including dialysis patients who are waitlisted for kidney transplantation [85–87].

Globally, these experimental findings provide a proof of concept for the usefulness of weight reduction and ACE inhibition interventions in the treatment of CKD in the obese. Studies showing a survival benefit of increased BMI in CKD patients, however, remain to be explained [88]. These findings limit our ability to make strong recommendations about the usefulness and the safety of weight reduction among individuals with more advanced stages of CKD. Lifestyle recommendations to reduce body weight in obese people at risk for CKD and in those with early CKD appear justified, particularly recommendations for the control of diabetes and hypertension. As the independent effect of obesity control on the incidence and progression of CKD is difficult to disentangle from the effects of hypertension and Type 2 diabetes, recommendation of weight loss in the minority of metabolically healthy and nonhypertensive obese patients remains unwarranted. These considerations suggest that a therapeutic approach to overweight and obesity in patients with advanced CKD or other significant comorbid conditions has to be pursued carefully, with proper considerations of the expected benefits and potential complications of weight loss over the life span of the individual patient.

## Conclusion

The worldwide epidemic of obesity affects the Earth's population in many ways. Diseases of the kidneys, including CKD, nephrolithiasis and kidney cancers are among the more insidious effects of obesity, but which nonetheless have wide ranging deleterious consequences, ultimately leading to significant excess morbidity and mortality and excess costs to individuals and the entire society. Population-wide interventions to control obesity could have beneficial effects in preventing the development, or delaying the progression of CKD. It is incumbent upon the entire healthcare community to devise long-ranging strategies toward improving the understanding of the links between obesity and kidney diseases, and to determine optimal strategies to stem the tide. The 2017 World Kidney Day is an important opportunity to increase education and awareness to that end.

## References

[1] Flegal KM, Kruszon-Moran D, Carroll MD, Fryar CD, Ogden CL (2016). Trends in obesity among adults in the United States, 2005 to 2014. *JAMA*.

[2] Forouzanfar MH, Alexander L, Anderson HR (2015). Global, regional, and national comparative risk assessment of 79 behavioural, environmental and occupational, and metabolic risks or clusters of risks in 188 countries, 1990–2013: a systematic analysis for the Global Burden of Disease Study 2013. *Lancet*.

[3] Cattaneo A, Monasta L, Stamatakis E (2010). Overweight and obesity in infants and pre-school children in the European Union: a review of existing data. *Obes. Rev.*.

[4] Olaya B, Moneta MV, Pez O (2015). Country-level and individual correlates of overweight and obesity among primary school children: a cross-sectional study in seven European countries. *BMC Public Health*.

[5] Subramanian SV, Perkins JM, Ozaltin E, Davey SG (2011). Weight of nations: a socioeconomic analysis of women in low- to middle-income countries. *Am. J. Clin. Nutr.*.

[6] Tsujimoto T, Sairenchi T, Iso H (2014). The dose-response relationship between body mass index and the risk of incident stage ≥3 chronic kidney disease in a general japanese population: the Ibaraki prefectural health study (IPHS). *J. Epidemiol.*.

[7] Elsayed EF, Sarnak MJ, Tighiouart H (2008). Waist-to-hip ratio, body mass index, and subsequent kidney disease and death. *Am. J. Kidney Dis.*.

[8] Pinto-Sietsma SJ, Navis G, Janssen WM, de Zeeuw D, Gans RO, de Jong PE (2003). A central body fat distribution is related to renal function impairment, even in lean subjects. *Am. J. Kidney Dis.*.

[9] Foster MC, Hwang SJ, Larson MG (2008). Overweight, obesity, and the development of stage 3 CKD: the Framingham Heart Study. *Am. J. Kidney Dis.*.

[10] Kramer H, Luke A, Bidani A, Cao G, Cooper R, McGee D (2005). Obesity and prevalent and incident CKD: the Hypertension Detection and Follow-Up Program. *Am. J. Kidney Dis.*.

[11] Chang A, Van HL, Jacobs DR (2013). Lifestyle-related factors, obesity, and incident microalbuminuria. the CARDIA (Coronary Artery Risk Development in Young Adults) study. *Am. J. Kidney Dis.*.

[12] Ejerblad E, Fored CM, Lindblad P, Fryzek J, McLaughlin JK, Nyren O (2006). Obesity and risk for chronic renal failure. *J. Am. Soc. Nephrol.*.

[13] Gelber RP, Kurth T, Kausz AT (2005). Association between body mass index and CKD in apparently healthy men. *Am. J. Kidney Dis.*.

[14] Lu JL, Molnar MZ, Naseer A, Mikkelsen MK, Kalantar-Zadeh K, Kovesdy CP (2015). Association of age and BMI with kidney function and mortality: a cohort study. *Lancet Diabetes Endocrinol.*.

[15] Munkhaugen J, Lydersen S, Wideroe TE, Hallan S (2009). Prehypertension, obesity, and risk of kidney disease: 20-year follow-up of the HUNT I study in Norway. *Am. J. Kidney Dis.*.

[16] Iseki K, Ikemiya Y, Kinjo K, Inoue T, Iseki C, Takishita S (2004). Body mass index and the risk of development of end-stage renal disease in a screened cohort. *Kidney Int.*.

[17] Vivante A, Golan E, Tzur D (2012). Body mass index in 1.2 million adolescents and risk for end-stage renal disease. *Arch. Intern. Med.*.

[18] Hsu C, McCulloch C, Iribarren C, Darbinian J, Go A (2006). Body mass index and risk for end-stage renal disease. *Ann. Intern. Med.*.

[19] Lu JL, Kalantar-Zadeh K, Ma JZ, Quarles LD, Kovesdy CP (2014). Association of body mass index with outcomes in patients with CKD. *J. Am. Soc. Nephrol.*.

[20] Thoenes M, Reil JC, Khan BV (2009). Abdominal obesity is associated with microalbuminuria and an elevated cardiovascular risk profile in patients with hypertension. *Vasc. Health Risk Manag.*.

[21] Kramer H, Gutierrez OM, Judd SE (2016). Waist circumference, body mass index, and ESRD in the REGARDS (Reasons for Geographic and Racial Differences in Stroke) study. *Am. J. Kidney Dis.*.

[22] Foster MC, Hwang SJ, Massaro JM (2011). Association of subcutaneous and visceral adiposity with albuminuria: the Framingham Heart Study. *Obesity (Silver Spring)*.

[23] Postorino M, Marino C, Tripepi G, Zoccali C (2009). Abdominal obesity and all-cause and cardiovascular mortality in end-stage renal disease. *J. Am. Coll. Cardiol.*.

[24] Kovesdy CP, Czira ME, Rudas A (2010). Body mass index, waist circumference and mortality in kidney transplant recipients. *Am. J. Transplant*.

[25] Scales CD, Smith AC, Hanley JM, Saigal CS (2012). Prevalence of kidney stones in the United States. *Eur. Urol.*.

[26] Curhan GC, Willett WC, Rimm EB, Speizer FE, Stampfer MJ (1998). Body size and risk of kidney stones. *J. Am. Soc. Nephrol.*.

[27] Taylor EN, Stampfer MJ, Curhan GC (2005). Obesity, weight gain, and the risk of kidney stones. *JAMA*.

[28] Bhaskaran K, Douglas I, Forbes H, dos-Santos-Silva I, Leon DA, Smeeth L (2014). Body-mass index and risk of 22 specific cancers: a population-based cohort study of 5.24 million UK adults. *Lancet*.

[29] Arnold M, Pandeya N, Byrnes G (2015). Global burden of cancer attributable to high body-mass index in 2012: a population-based study. *Lancet Oncol.*.

[30] Renehan AG, Tyson M, Egger M, Heller RF, Zwahlen M (2008). Body-mass index and incidence of cancer: a systematic review and meta-analysis of prospective observational studies. *Lancet*.

[31] Bluher M (2010). The distinction of metabolically ‘healthy’ from ‘unhealthy’ obese individuals. *Curr. Opin. Lipidol.*.

[32] Sharma K (2009). The link between obesity and albuminuria: adiponectin and podocyte dysfunction. *Kidney Int.*.

[33] Wolf G, Ziyadeh FN (2006). Leptin and renal fibrosis. *Contrib. Nephrol.*.

[34] Ellington AA, Malik AR, Klee GG (2007). Association of plasma resistin with glomerular filtration rate and albuminuria in hypertensive adults. *Hypertension*.

[35] Bastard JP, Maachi M, Lagathu C (2006). Recent advances in the relationship between obesity, inflammation, and insulin resistance. *Eur. Cytokine Netw.*.

[36] Furukawa S, Fujita T, Shimabukuro M (2004). Increased oxidative stress in obesity and its impact on metabolic syndrome. *J. Clin. Invest.*.

[37] Ruan XZ, Varghese Z, Moorhead JF (2009). An update on the lipid nephrotoxicity hypothesis. *Nat. Rev. Nephrol.*.

[38] Ruster C, Wolf G (2013). The role of the renin-angiotensin-aldosterone system in obesity-related renal diseases. *Semin. Nephrol.*.

[39] Oterdoom LH, de Vries AP, Gansevoort RT, de Jong PE, Gans RO, Bakker SJ (2007). Fasting insulin modifies the relation between age and renal function. *Nephrol. Dial. Transplant.*.

[40] Reaven GM (1988). Banting lecture 1988: role of insulin resistance in human disease. *Diabetes*.

[41] Kambham N, Markowitz GS, Valeri AM, Lin J, D'Agati VD (2001). Obesity-related glomerulopathy: an emerging epidemic. *Kidney Int.*.

[42] de Vries AP, Ruggenenti P, Ruan XZ (2014). Fatty kidney: emerging role of ectopic lipid in obesity-related renal disease. *Lancet Diabetes Endocrinol.*.

[43] Foster MC, Hwang SJ, Porter SA, Massaro JM, Hoffmann U, Fox CS (2011). Fatty kidney, hypertension, and chronic kidney disease: the Framingham Heart Study. *Hypertension*.

[44] Henegar JR, Bigler SA, Henegar LK, Tyagi SC, Hall JE (2001). Functional and structural changes in the kidney in the early stages of obesity. *J. Am. Soc. Nephrol.*.

[45] Knight SF, Quigley JE, Yuan J, Roy SS, Elmarakby A, Imig JD (2008). Endothelial dysfunction and the development of renal injury in spontaneously hypertensive rats fed a high-fat diet. *Hypertension*.

[46] Tsuboi N, Utsunomiya Y, Kanzaki G (2012). Low glomerular density with glomerulomegaly in obesity-related glomerulopathy. *Clin. J. Am. Soc. Nephrol.*.

[47] Ribstein J, du CG, Mimran A (1995). Combined renal effects of overweight and hypertension. *Hypertension*.

[48] Maalouf NM, Sakhaee K, Parks JH, Coe FL, Adams-Huet B, Pak CY (2004). Association of urinary pH with body weight in nephrolithiasis. *Kidney Int.*.

[49] Lemann J, Pleuss JA, Worcester EM, Hornick L, Schrab D, Hoffmann RG (1996). Urinary oxalate excretion increases with body size and decreases with increasing dietary calcium intake among healthy adults. *Kidney Int.*.

[50] Siener R, Glatz S, Nicolay C, Hesse A (2004). The role of overweight and obesity in calcium oxalate stone formation. *Obes. Res.*.

[51] Taylor EN, Stampfer MJ, Curhan GC (2005). Diabetes mellitus and the risk of nephrolithiasis. *Kidney Int.*.

[52] Klisic J, Hu MC, Nief V (2002). Insulin activates Na(+)/H(+) exchanger 3: biphasic response and glucocorticoid dependence. *Am J. Physiol. Renal Physiol.*.

[53] Chobanian MC, Hammerman MR (1987). Insulin stimulates ammoniagenesis in canine renal proximal tubular segments. *Am. J. Physiol.*.

[54] Daudon M, Lacour B, Jungers P (2006). Influence of body size on urinary stone composition in men and women. *Urol. Res.*.

[55] Sinha MK, Collazo-Clavell ML, Rule A (2007). Hyperoxaluric nephrolithiasis is a complication of Roux-en-Y gastric bypass surgery. *Kidney Int.*.

[56] Calle EE, Kaaks R (2004). Overweight, obesity and cancer: epidemiological evidence and proposed mechanisms. *Nat. Rev. Cancer*.

[57] Dalamaga M, Diakopoulos KN, Mantzoros CS (2012). The role of adiponectin in cancer: a review of current evidence. *Endocr. Rev.*.

[58] Lamas O, Marti A, Martinez JA (2002). Obesity and immunocompetence. *Eur. J. Clin. Nutr.*.

[59] Lim C, Savan R (2014). The role of the IL-22/IL-22R1 axis in cancer. *Cytokine Growth Factor Rev.*.

[60] Grivennikov SI, Greten FR, Karin M (2010). Immunity, inflammation, and cancer. *Cell*.

[61] Kovesdy CP, Anderson JE, Kalantar-Zadeh K (2007). Paradoxical association between body mass index and mortality in men with CKD not yet on dialysis. *Am. J. Kidney Dis.*.

[62] Kalantar-Zadeh K, Kuwae N, Wu DY (2006). Associations of body fat and its changes over time with quality of life and prospective mortality in hemodialysis patients. *Am. J. Clin. Nutr.*.

[63] Beddhu S, Pappas LM, Ramkumar N, Samore M (2003). Effects of body size and body composition on survival in hemodialysis patients. *J. Am. Soc. Nephrol.*.

[64] Curtis JP, Selter JG, Wang Y (2005). The obesity paradox. body mass index and outcomes in patients with heart failure. *Arch. Intern. Med.*.

[65] Wilson DO, Rogers RM, Wright EC, Anthonisen NR (1989). Body weight in chronic obstructive pulmonary disease. The National Institutes of Health Intermittent Positive-Pressure Breathing Trial. *Am. Rev. Respir. Dis*.

[66] Escalante A, Haas RW, del Rincón I (2005). Paradoxical effect of body mass index on survival in rheumatoid arthritis: role of comorbidity and systemic inflammation. *Arch. Intern. Med.*.

[67] Kalantar-Zadeh K, Kilpatrick RD, Kuwae N, Wu DY (2005). Reverse epidemiology: a spurious hypothesis or a hardcore reality?. *Blood Purif.*.

[68] Noori N, Kopple JD, Kovesdy CP (2010). Mid-arm muscle circumference and quality of life and survival in maintenance hemodialysis patients. *Clin. J. Am. Soc. Nephrol.*.

[69] Dekker FW, de MR, van Dijk PC, Zoccali C, Jager KJ (2008). Survival analysis: time-dependent effects and time-varying risk factors. *Kidney Int.*.

[70] Snyder JJ, Foley RN, Gilbertson DT, Vonesh EF, Collins AJ (2003). Body size and outcomes on peritoneal dialysis in the United States. *Kidney Int.*.

[71] Lee PS, Sampath K, Karumanchi SA (2009). Plasma gelsolin and circulating actin correlate with hemodialysis mortality. *J. Am. Soc. Nephrol.*.

[72] Horwich TB, Fonarow GC, Hamilton MA, MacLellan WR, Woo MA, Tillisch JH (2001). The relationship between obesity and mortality in patients with heart failure. *J. Am. Coll. Cardiol.*.

[73] Stenvinkel P, Marchlewska A, Pecoits-Filho R (2004). Adiponectin in renal disease. relationship to phenotype and genetic variation in the gene encoding adiponectin. *Kidney Int.*.

[74] Mohamed-Ali V, Goodrick S, Bulmer K, Holly JM, Yudkin JS, Coppack SW (1999). Production of soluble tumor necrosis factor receptors by human subcutaneous adipose tissue *in vivo*. *Am. J. Physiol.*.

[75] Rauchhaus M, Coats AJ, Anker SD (2000). The endotoxin-lipoprotein hypothesis. *Lancet*.

[76] Jandacek RJ, Anderson N, Liu M, Zheng S, Yang Q, Tso P (2005). Effects of yo-yo diet, caloric restriction, and olestra on tissue distribution of hexachlorobenzene. *Am. J. Physiol. Gastrointest. Liver Physiol.*.

[77] Kramer HJ, Saranathan A, Luke A (2006). Increasing body mass index and obesity in the incident ESRD population. *J. Am. Soc. Nephrol.*.

[78] Postorino M, Mancini E, D'Arrigo G (2016). Body mass index trend in haemodialysis patients: the shift of nutritional disorders in two Italian regions. *Nephrol. Dial. Transplant.*.

[79] World Health Organisation (2009). 2008–2013 Action Plan for the Global Strategy for the Prevention and Control of Noncommunicable Diseases. http://www.apps.who.int/iris/bitstream/10665/44009/1/9789241597418_eng.pdf.

[80] O'Donoghue DJ, Stevens PE (2012). A decade after the KDOQI CKD/guidelines: a perspective from the United Kingdom. *Am. J. Kidney Dis.*.

[81] Bolignano D, Zoccali C (2013). Effects of weight loss on renal function in obese CKD patients: a systematic review. *Nephrol. Dial. Transplant.*.

[82] Chang Y, Ryu S, Choi Y (2016). Metabolically healthy obesity and development of chronic kidney disease: a cohort study. *Ann. Intern. Med.*.

[83] Wing RR, Bolin P, Brancati FL (2013). Cardiovascular effects of intensive lifestyle intervention in type 2 diabetes. *N. Engl. J. Med.*.

[84] Mallamaci F, Ruggenenti P, Perna A (2011). ACE inhibition is renoprotective among obese patients with proteinuria. *J. Am. Soc. Nephrol.*.

[85] Friedman AN, Wolfe B (2016). Is bariatric surgery an effective treatment for type II diabetic kidney disease?. *Clin. J. Am. Soc. Nephrol.*.

[86] Chang AR, Chen Y, Still C (2016). Bariatric surgery is associated with improvement in kidney outcomes. *Kidney Int.*.

[87] Jamal MH, Corcelles R, Daigle CR (2015). Safety and effectiveness of bariatric surgery in dialysis patients and kidney transplantation candidates. *Surg. Obes. Relat. Dis.*.

[88] Ahmadi SF, Zahmatkesh G, Ahmadi E (2015). Association of body mass index with clinical outcomes in non-dialysis-dependent chronic kidney disease: a systematic review and meta-analysis. *Cardiorenal. Med.*.

